# Australian Women’s Intentions and Psychological Outcomes Related to Breast Density Notification and Information: A Randomized Clinical Trial

**DOI:** 10.1001/jamanetworkopen.2022.16784

**Published:** 2022-06-16

**Authors:** Hankiz Dolan, Kirsten McCaffery, Nehmat Houssami, Erin Cvejic, Meagan Brennan, Jolyn Hersch, Melanie Dorrington, Angela Verde, Lisa Vaccaro, Brooke Nickel

**Affiliations:** 1 Wiser Healthcare, Sydney School of Public Health, Faculty of Medicine and Health, The University of Sydney, Sydney, Australia; 2 Sydney Health Literacy Lab, Sydney School of Public Health, Faculty of Medicine and Health, The University of Sydney, Sydney, Australia; 3 The Daffodil Centre, The University of Sydney, a joint venture with Cancer Council NSW, Sydney, Australia; 4 University of Notre Dame Australia, School of Medicine Sydney, Sydney, Australia; 5 Westmead Breast Cancer Institute, Westmead Hospital, Sydney, Sydney, Australia; 6 Bungendore Medical Centre, Bungendore, Australia; 7 Breast Cancer Network Australia, Melbourne, Australia; 8 Health Consumers New South Wales, Sydney, Australia; 9 Discipline of Behavioural and Social Sciences in Health, Sydney School of Health Sciences, Faculty of Medicine and Health, The University of Sydney, Sydney, Australia

## Abstract

**Importance:**

Whether the benefits of notifying women about breast density outweigh the potential harms to inform current and future mammogram screening practice remains unknown.

**Objective:**

To assess the effect of mammographic breast density notification and information provision on women’s intention to seek supplemental screening and psychological outcomes.

**Design, Setting, and Participants:**

A 3-arm online randomized clinical trial was conducted from August 10 to 31, 2021. Data analysis was conducted from September 1 to October 20, 2021. Participants included Australian residents identifying as female, aged between 40 and 74 years, with no history of breast cancer who were residing in jurisdictions without existing breast density notification with screening mammograms.

**Interventions:**

Women were randomized to receive 1 of the following hypothetical breast screening test result letters: screening mammogram result letter without breast density messaging (control), screening mammogram result letter with breast density messaging and an existing density information letter taken from a screening service in Australia (intervention 1), and screening mammogram result letter with breast density messaging and a health literacy–sensitive version of the letter adapted for people with lower health literacy (intervention 2).

**Main Outcomes and Measures:**

Primary outcomes were intention to seek supplemental screening; feeling anxious (uneasy, worried, or nervous), informed, or confused; and having breast cancer worry.

**Results:**

A total of 1420 Australian women were randomized and included in the final analysis. The largest group consisted of 603 women aged 60 to 74 years (42.5%). Compared with the control cohort (n = 480), women who received density notification via intervention 1 (n = 470) and intervention 2 (n = 470) reported a significantly higher intention to seek supplemental screening (0.8% vs 15.6% and 14.2%; *P* < .001) and feeling anxious (14.2% vs 49.4% and 48.5%; *P* < .001), confusion (7.8% vs 24.0% and 23.6%; *P* < .001), and worry about breast cancer (quite/very worried: 6.9% vs 17.2% and 15.5%; *P* < .001). There were no statistically significant differences in these outcomes between the 2 intervention groups.

**Conclusions and Relevance:**

In this randomized clinical trial, breast density notification and information integrated with screening mammogram results increased women’s intention to seek supplemental screening and made women feel anxious, confused, or worried about breast cancer. These findings have relevance and implications for mammogram screening services and policy makers considering whether and, if so, how best to implement widespread notification of breast density as part of mammography screening.

**Trial Registration:**

ACTRN12621000253808

## Key Points

**Question:**

What is the effect of mammographic breast density notification and information provision on women’s intention to seek supplemental screening and self-reported psychological outcomes?

**Findings:**

In this randomized clinical trial of 1420 women living in Australian jurisdictions without widespread breast density notification, women who viewed a hypothetical screening mammogram results letter with breast density notification and information were significantly more likely to report intention to seek supplemental screening and feeling anxious, uneasy, confused, and worried about developing cancer.

**Meaning:**

The findings of this trial indicate that more evidence on overall benefits and harms of notification, as well as adequate and equitable service planning and communication strategies, is warranted to inform decisions on widespread breast density notification.

## Teaser

This randomized clinical trial evaluates the response to notification of breast density and provision of information for women with increased breast density following screening mammograms.

## Introduction

Mammographic breast density is one of several independent risk factors for breast cancer.^1^ Dense breast tissue can obscure a tumor when examining a mammogram image because both appear white and therefore can lead to reduced mammographic sensitivity and an increased chance of being diagnosed as breast cancer between routine screening mammograms.^2,3^ Although there have been investigations into the interactions of some drugs with breast density,^4^ the density remains relatively nonmodifiable, unlike other risk factors, such as body mass index and alcohol consumption.^5^ It is estimated that a quarter to half of the population of women who are of breast screening age (40-75 years) have heterogeneously and extremely dense breasts.^6,7^ The estimate varies depending on the age of the screening population, because breast density usually decreases with age,^8^ and how breast density is measured and classified.^6,7^

Internationally, one of the major movements regarding breast density notification was the introduction of federal legislation in 2020 in the US mandating breast density notification to women following mammograms.^9^ Since then, there has been a continuous stream of research on the outcomes of notification in women, health care professionals, and the health system, and communication strategies to improve relevant outcomes.^10,11,12,13^ There has been much discussion around the management options for women after they are notified of having dense breasts and the benefits and harms of supplemental screening, such as ultrasonography or magnetic resonance imaging, in addition to routine mammographic screening.^14^ The option of supplemental screening has been controversial, with a lack of evidence in terms of long-term health benefits as well as unintended adverse consequences, such as widening health inequality, including reduced access of women of other racial and ethnic populations to supplemental screening.^15^ The readability, accessibility, and acceptability of existing breast density educational and informational materials were also scrutinized, often highlighting the need for lower health literacy (HL)–sensitive material development.^16,17,18^ In other countries, there has been increased discussion and consumer advocacy around density notification; however, no legislation or mandated reporting is enforced.

In Australia, asymptomatic women aged 50 to 74 years are actively invited to participate in free screening mammograms every 2 years, with the free program being open to those aged 40 to 49 years or 75 years and older without invitation.^19^ However, except for the state of Western Australia (WA), breast density is neither measured nor reported by the publicly funded breast screening programs. Given this, most Australian women of breast screening age do not receive breast density notification.

There is currently limited evidence on the outcomes associated with informing women about their breast density. Similarly, there is a lack of research into how attention to HL in written information on breast density might further affect women’s understanding of breast density and their follow-up intentions and behaviors. Therefore, the present study used a randomized design to assess how breast density notification and different formats of information provision affect women’s screening intentions and psychological outcomes using a hypothetical clinical scenario. The findings may provide a foundation to directly inform policy makers and lay the groundwork for subsequent clinical studies into breast density notification and the psychological and behavioral consequences.

## Methods

### Study Design

This was an online randomized clinical trial and followed the Consolidated Standards of Reporting Trials (CONSORT) reporting guideline for randomized clinical trials. The trial protocol and statistical analysis plan are available in Supplement 1. This study received ethical approval from The University of Sydney. Participants were recruited through an independent social research company (Dynata), which has an extensive panel of participants whose demographic characteristics align closely with those of the national population. Potential participants were directed to a study landing webpage, where they were able to view the information statement and gave consent in an online form before proceeding to the screening questions and main questionnaire (eAppendix in Supplement 2). Participation is incentivized using points, which can be redeemed for gift vouchers. The data were collected from August 10 to 31, 2021, and data analysis was performed from September 1 to October 20, 2021. An online survey platform (Qualtrics) was used to administer the questionnaire.

Australian residents identifying as female, aged 40 to 74 years, with no personal history of breast cancer or ductal carcinoma in situ, and who reside outside of WA were eligible to take part in the study. Based on the educational attainment distribution of women aged 35 to 69 years in the Australian population according to 2016 census data,^20^ we quota-sampled to recruit 30% of women with a bachelor’s degree or above, 30% of women with a diploma or certificate, and 40% of women without tertiary education qualifications.

After completing sociodemographic, including reporting of White, Torres Strait Islander, and Aboriginal race and ethnicity, and baseline questions, participants were presented with a hypothetical scenario of going for routine mammography screening for breast cancer. They were then randomized to be presented with 1 of 3 example letters about the mammography results. Randomization was performed by the survey platform using a random number generator. Participants and the survey administrators were blinded to the group allocation at the time of the randomization.

### Procedures

#### Control

Participants were shown a generic mammography results letter reporting that no breast cancer could be seen on the mammogram. This document was adapted from standard normal mammography result letters from New South Wales and WA.

#### Intervention 1

Participants in the WA letter cohort were shown the same generic mammography letter as the controls with an additional notification that the screening mammogram showed their breasts are dense. The wording of this message was adapted from the existing standard mammography letter used in BreastScreen WA public screening services in notifying women of their breast density. Along with this letter, the WA letter group was presented with an information pamphlet on breast density currently used in WA (omitting identifying headers and footers including logos and contact information).^21^ This letter had a readability level of 12.2 (measured using Microsoft Word Flesch-Kincaid grade level test, which is well above the recommended reading level of grade 8; a lower grade reading score means that the text is easier to read and aiming for a grade 8 readability score is advised for most audiences.).^22,23^

#### Intervention 2

Participants were shown the same mammography results letter as the WA letter group, with a HL-adapted information pamphlet on breast density, which was developed by the research team. Adaptation involved the use of simpler language, removing jargon, reducing sentence length and complexity, and use of the active voice. This process was achieved using the publicly available Sydney Health Literacy Lab Health Literacy Editor.^24^ The letter had a readability level of grade 7.8 on the Microsoft Word Flesch-Kincaid test (with grade ≤8 recommended for average readers).

To maximize the likelihood of participants reading the example results letter and the information about breast density, minimum time requirements before being able to proceed roughly based on word length were added to the mammography result letters (30 seconds), WA letter (40 seconds), and the HL-sensitive letter (60 seconds). Copies of the control and intervention materials are provided in the eAppendix of Supplement 2.

### Outcomes

We used a combination of previously published, validated, and study-specific self-developed questions by the multidisciplinary study team in collecting the baseline and outcome data. Immediately before the intervention, participants completed sociodemographic characteristic questions as presented in Table 1. Participants then answered questions related to general health,^25^ personal and family cancer history,^28^ and cancer worry.^29^ Overall well-being in the last 2 weeks of the study was measured using the World Health Organization–5 tool (range, 0 [worst possible well-being] to 100 [best possible well-being]).^27^ Health literacy was measured using the widely used single-item HL screener.^26^

**Table 1.** Sample Characteristics in 1420 Women

Variable	No. (%)		
	Control (n = 480)	WA letter (n = 470)	HL letter (n = 470)
Age, y			
40-49	143 (29.8)	118 (25.1)	135 (28.7)
50-59	137 (28.5)	140 (29.8)	144 (30.6)
60-74	200 (41.7)	212 (45.1)	191 (40.6)
State			
New South Wales	144 (30.0)	135 (28.7)	153 (32.6)
Victoria	149 (31.0)	135 (28.7)	115 (24.5)
Australian Capital Territory	4 (0.8)	8 (1.7)	6 (1.3)
Queensland	108 (22.5)	108 (23.0)	129 (27.4)
South Australia	61 (12.7)	48 (10.2)	46 (9.8)
Northern Territory	4 (0.8)	2 (0.4)	1 (0.2)
Tasmania	10 (2.1)	34 (7.2)	20 (4.3)
Educational level			
Bachelor’s degree or above	147 (30.6)	135 (28.7)	141 (30.0)
Diploma or certificate	144 (30.0)	132 (28.1)	155 (33.0)
High school or below	189 (39.4)	203 (43.2)	174 (37.0)
Employment status			
Permanent/ongoing/fixed-term contract/on paid leave	141 (29.4)	148 (31.5)	145 (30.9)
Casual or temporary/self-employed	66 (13.8)	73 (15.5)	67 (14.3)
Unemployed/not working	273 (56.9)	249 (53.0)	258 (54.9)
Household income (AUD)			
$50 000 or less	231 (48.1)	218 (46.4)	217 (46.2)
Between $50 000 and $100 000	125 (26.0)	154 (32.8)	136 (28.9)
More than $100 000	124 (25.8)	98 (20.9)	117 (24.9)
Relationship			
Married/de-facto/in a relationship	283 (59.0)	304 (64.7)	296 (63.0)
Single and never married	85 (17.7)	66 (14.0)	71 (15.1)
Widowed/divorced/separated	112 (23.3)	100 (21.3)	103 (21.9)
No. of children			
0	133 (27.7)	115 (24.5)	138 (29.4)
1-4	345 (71.9)	355 (75.5)	328 (69.7)
Prefer not to say	2 (0.4)	0	4 (0.9)
Aboriginal and/or Torres Strait Islander origin			
Aboriginal origin	9 (1.9)	5 (1.1)	7 (1.5)
Torres Strait Islander origin	0	0	0
Both Aboriginal and Torres Strait Islander origin	0	3 (0.6)	0
Neither	471 (98.1)	462 (98.3)	463 (98.5)
Birthplace			
Australia	350 (72.9)	351 (74.7)	353 (75.1)
Overseas	130 (27.1)	119 (25.3)	117 (24.9)
Main language spoken at home			
English	456 (95.0)	446 (94.9)	442 (94)
Other	24 (5.0)	24 (5.1)	28 (6.0)
Private health insurance			
Yes	240 (50.0)	244 (51.9)	262 (55.7)
No	240 (50.0)	226 (48.1)	208 (44.3)
Personal cancer history (excluding breast cancer)			
Yes	33 (6.9)	29 (6.2)	44 (9.4)
No	446 (92.9)	440 (93.6)	423 (90.0)
Do not know	1 (0.2)	1 (0.2)	3 (0.6)
Family history of cancer (parents, siblings, or children)			
Yes	247 (51.5)	252 (53.6)	252 (53.6)
No	224 (46.7)	213 (45.3)	208 (44.3)
Do not know	9 (1.9)	5 (1.1)	10 (2.1)
Family history of breast cancer			
Yes	71 (14.8)	84 (17.9)	69 (14.7)
No	409 (85.2)	386 (82.1)	401 (85.3)
Self-reported general health^a^			
Excellent	27 (5.6)	23 (4.9)	31 (6.6)
Very good	143 (29.8)	136 (28.9)	129 (27.4)
Good	185 (38.5)	189 (40.2)	187 (39.8)
Fair	108 (22.5)	99 (21.1)	95 (20.2)
Poor	17 (3.5)	23 (4.9)	28 (6.0)
Prior knowledge of breast density			
Yes	197 (41.0)	222 (47.2)	220 (46.8)
No	283 (59.0)	248 (52.8)	250 (53.2)
If yes, do you have dense breasts?			
Yes	56 (28.4)	49 (22.1)	59 (26.8)
No	63 (32.0)	84 (37.8)	65 (29.5)
Do not know	78 (39.6)	89 (40.1)	96 (43.6)
Mammogram history			
Yes	357 (74.4)	358 (76.2)	355 (75.5)
No	123 (25.6)	112 (23.8)	115 (24.5)
Health literacy^b^			
Adequate	443 (92.3)	431 (91.7)	425 (90.4)
Not adequate	37 (7.7)	39 (8.3)	45 (9.6)
WHO-5, mean (SD)^c^	53.55 (25.27)	54.25 (25.62)	50.89 (24.87)

Abbreviation: WHO, World Health Organization.

^a^ Measured using a validated single item.^25^

^b^ Measured using a validated single item. Never/rarely is adequate, and sometimes/often/always is inadequate.^26^

^c^ A score of 0 represents the worst possible well-being and 100 represents the best possible well-being.^27^

Primary outcome measures were screening intentions, including intention to seek supplemental screening, feeling anxious (uneasy, worried, or nervous), feeling informed or confused after receiving the letter,^28^ and having breast cancer worry.^29^ Secondary outcomes included intention to speak to a general practitioner (GP); breast density knowledge about increased cancer risk, prevalence, masking effect, and breast density decreasing with age; and cancer risk perception.^28,30^ The eAppendix in Supplement 2 includes the full questionnaire.

### Statistical Analysis

The required sample size was 1398, which would have 80% power to detect a moderate effect size (0.25) at an adjusted α value of 0.025 to account for multiple comparisons between either intervention arm and the control arm. Descriptive statistics (frequency and relative frequency for categorical variables, mean [SD] for continuous variables) of participant sociodemographic and health characteristics, as well as primary and secondary outcome measures, were calculated using SPSS, version 26 software (IBM Corp). Between-group comparisons of categorical variables were analyzed using 2-tailed χ^2^ tests. Multinomial logistic regression was used to analyze change in cancer worry from baseline (ie, reduction, no change, or increase after receiving results letter compared with baseline) between groups.

## Results

### Sociodemographic and Health Characteristics

**Figure 1.**
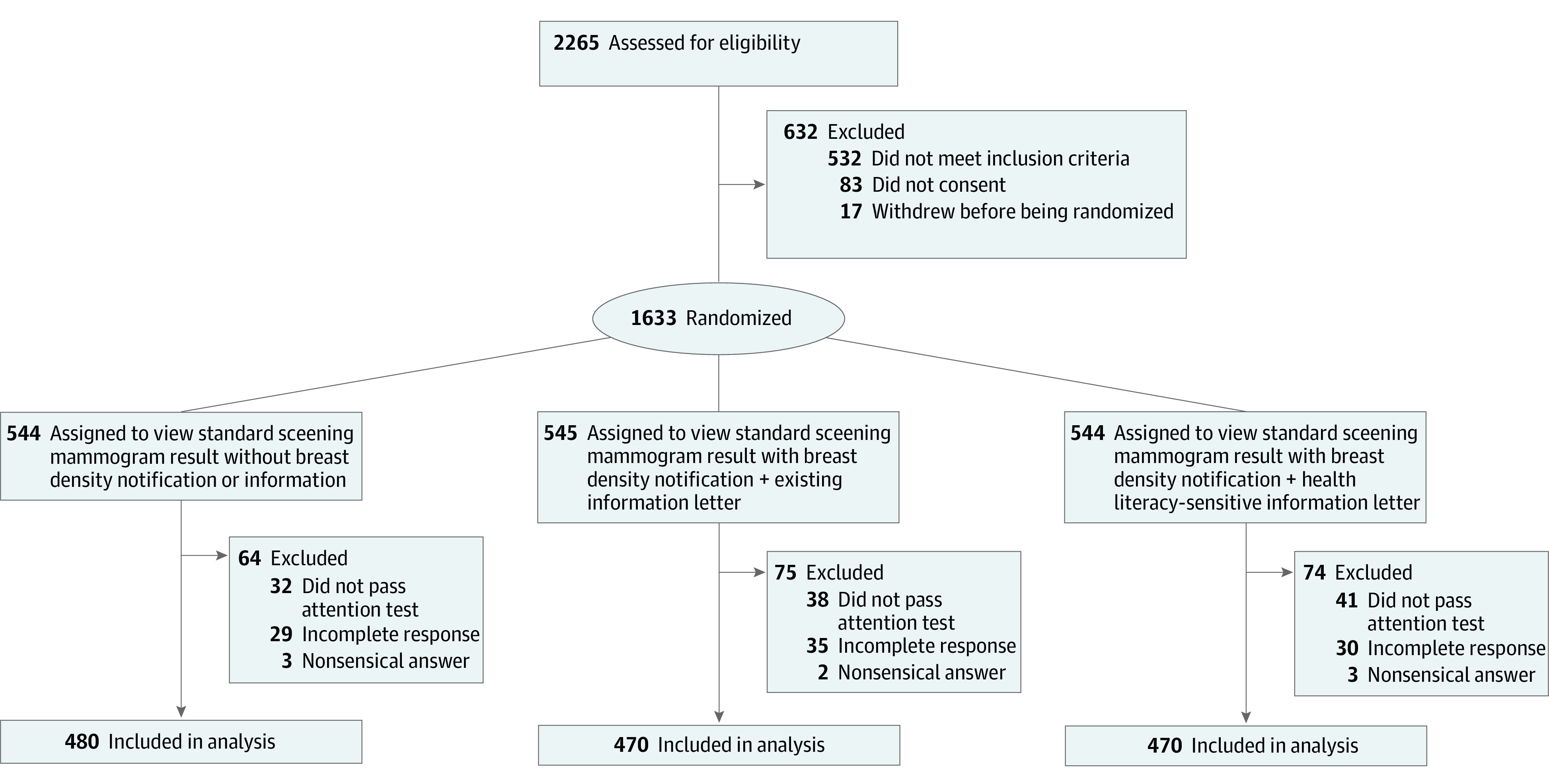
Flow Diagram

A total of 2265 respondents accessed the online study, with 1420 included in the final analysis (control: n = 480, WA letter: n = 470, HL letter: n = 470) (Figure 1). The sample characteristics by study arms are reported in Table 1. The largest group consisted of 603 women aged 60 to 74 years (42.5%). Most participants lived in New South Wales, Victoria, or Queensland (1176 [82.8%]), were born in Australia (1054 [74.2%]), and spoke English as a main language at home (1344 [94.6%]). Compared with population statistics for women of the same age group,^20^ the sample was skewed toward higher unemployment, lower household income, and a higher proportion being born in Australia and speaking English as the main language at home. There were 7.5% (n = 106) of the participants who had a personal history of cancer and 15.8% (n = 224) who had a family history of breast cancer, and 73.9% (n = 1050) rated their health as good to excellent. Forty percent (n = 566) of participants completed high school level education or less, with 91.5% (n = 1299) reporting adequate HL.

### Primary Outcomes

#### Intention to Undergo Supplemental Screening

**Figure 2.**
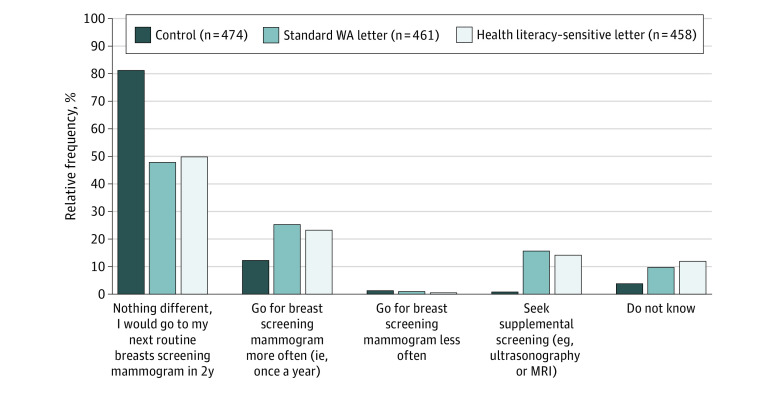
Screening Intentions After Reading the Letters Responses from 27 participants were excluded due to a technical error allowing for responses in more than one category without instruction to do so. MRI indicates magnetic resonance imaging; WA, Western Australia.

Compared with the control group, both intervention groups had a significantly higher proportion of women intending to seek supplemental screening (control: 4 [0.8%], WA letter: 72 [15.6%], HL letter: 65 [14.2%]; *P* < .001) and undergo screening mammography more often (control: 59 [12.4%], WA letter: 117 [25.4%], HL letter: 107 [23.4%]; *P* < .001). There were no significant between-group differences in screening intentions between the 2 intervention groups for supplemental screening (WA letter: 15.6% vs HL letter: 14.2%; *P* = .67) and increased screening frequency (WA letter: 25.4% vs 23.4% [HL letter]; *P* = .67) (Figure 2 and Table 2).

**Table 2.** Primary and Secondary Outcomes

Variable	No. (%)			*P* value	
	Control (n = 480)	WA letter (n = 470)	HL letter (n = 470)	Between group differences	Between WA and HL letter
Screening intention^a^					
Nothing different, I would go to my next routine breast screening mammogram in 2 y	385 (81.2)	221 (47.9)	227 (49.6)	<.001	.67
Go for breast screening mammogram more often, ie, once a year	59 (12.4)	117 (25.4)	107 (23.4)		
Go for breast screening mammogram less often	7 (1.5)	5 (1.1)	3 (0.7)		
Seek supplemental screening, such as ultrasonography or MRI	4 (0.8)	72 (15.6)	65 (14.2)		
Do not know	19 (3.9)	46 (9.7)	56 (12.2)		
Feeling anxious (uneasy, worried, nervous)					
Strongly agree	21 (4.4)	44 (9.4)	39 (8.3)	<.001	.89
Agree	47 (9.8)	188 (40.0)	189 (40.2)		
Disagree	238 (49.6)	201 (42.8)	200 (42.6)		
Strongly disagree	174 (36.3)	37 (7.9)	42 (8.9)		
Feeling informed^b^					
Strongly agree	163 (33.9)	129 (27.4)	132 (28.1)	.01	.67
Agree	290 (60.4)	304 (64.7)	310 (65.9)		
Strongly disagree or disagree	27 (5.6)	37 (7.9)	28 (5.9)		
Feeling confused					
Strongly agree	6 (1.3)	21 (4.5)	22 (4.7)	<.001	.99
Agree	31 (6.5)	92 (19.6)	89 (18.9)		
Disagree	232 (48.3)	275 (58.5)	279 (59.4)		
Strongly disagree	211 (43.9)	82 (17.4)	80 (17.0)		
Breast cancer worry (after intervention)					
Not worried at all	263 (54.8)	121 (25.7)	133 (28.3)	<.001	.55
A bit worried	184 (38.3)	268 (57.0)	264 (56.2)		
Quite worried	20 (4.2)	50 (10.6)	51 (10.9)		
Very worried	13 (2.7)	31 (6.6)	22 (4.7)		
Intention to speak with a primary care practitioner					
Yes	138 (28.7)	318 (67.7)	309 (65.7)	<.001	.71
No	300 (62.5)	86 (18.3)	96 (20.4)		
Do not know	42 (8.8)	66 (14.0)	65 (13.8)		
Knowledge, percentage of women with dense breasts					
Incorrect answer or do not know	370 (77.1)	275 (58.5)	200 (42.6)	<.001	<.001
Correct answer	110 (22.9)	195 (41.5)	270 (57.4)		
Knowledge, breast density and breast cancer risk					
Incorrect answer or do not know	392 (81.7)	372 (79.1)	196 (41.7)	<.001	<.001
Correct answer	88 (18.3)	98 (20.9)	274 (58.3)		
Knowledge, dense breasts masking effect on mammogram					
Incorrect answer or do not know	291 (60.6)	94 (20.)	134 (28.5)	<.001	.001
Correct answer	189 (39.4)	376 (80.0)	336 (71.5)		
Knowledge, breast density decreases with age					
Incorrect answer or do not know	397 (82.7)	183 (38.9)	241 (51.3)	<.001	<.001
Correct answer	83 (17.3)	287 (61.1)	229 (48.7)		
Breast cancer risk perception					
Below average	129 (26.9)	86 (18.3)	105 (22.3)	<.001	.27
Average	339 (70.6)	342 (72.8)	321 (68.3)		
Above average	12 (2.5)	42 (8.9)	44 (9.4)		

Abbreviations: HL, health literacy; MRI, magnetic resonance imaging; WA, Western Australia.

^a^ Responses from 27 participants were excluded due to a technical error allowing for responses in more than one category without instruction to do so.

^b^ Strongly disagree and disagree response categories were combined due to small relative frequencies.

#### Psychological Outcomes

Compared with the control group, significantly higher proportions of participants in the intervention groups agreed or strongly agreed that the letter made them feel anxious (uneasy, worried, nervous) (control: 68 [14.2%], WA letter: 232 [49.4%], HL letter: 228 [48.5%]; *P* < .001). There was no significant difference between the 2 intervention groups (WA letter: 49.4% vs HL letter 48.5%; *P* = .89) (Table 2).

Compared with the control group, a similar proportion of participants in the intervention groups agreed or strongly agreed that the letter made them feel informed to make decisions regarding their breast health (control: 453 [94.4%], WA letter: 433 [92.1%], HL letter: 442 [94.1%]; *P* = .56). There was no significant difference between the 2 intervention groups (WA letter: 92.1% vs HL letter: 94.1%; *P* = .67).

Compared with the control group, a significantly higher proportion of participants in the intervention groups agreed or strongly agreed that the letter made them feel confused about what to do regarding their breast health (control: 37 [7.8%], WA letter: 113 [24.0%], HL letter: 111 [23.6%]; *P* < .001). There was no significant difference between the 2 intervention groups (WA letter: 24.0% vs HL letter: 23.6%; *P* = .99).

Compared with the control group, significantly higher proportions of participants in the intervention groups reported feeling quite or very worried about developing breast cancer (control: 33 [6.9%], WA letter: 81 [17.2%], HL letter: 73 [15.5%]; *P* < .001). There was no significant difference between the 2 intervention groups (WA letter: 17.2% vs HL letter: 15.5%; *P* = .55). Participants in the intervention groups were also more likely to indicate an increase in cancer worry from baseline, compared with the control group. Relative to an outcome of no change, participants were more likely to indicate increased cancer worry if they received the WA letter (relative risk, 4.54; 95% CI, 2.87-7.18; *P* < .001) or the HL letter (RR, 4.61; 95% CI, 2.91-7.29; *P* < .001), compared with the control group. There was no evidence of a difference between the intervention groups (eTable in Supplement 2).

### Secondary Outcomes

Compared with the control participants, significantly higher proportions of participants in the intervention groups reported planning to talk to a GP about the letter (control: 28.7%, WA letter: 67.7%, HL letter: 65.7%; *P* < .001). There was no significant difference between the WA and HL letter intervention groups (WA letter: 67.7% vs HL letter: 65.7%; *P* = .71).

Regarding breast density knowledge, compared with the control group, both the intervention groups had significantly higher proportions of participants correctly answering all 4 questions about breast density (control: 24.5%, WA letter: 50.9%, HL letter: 59.0%; *P* < .001). Compared with participants who received the WA letter, a significantly higher proportion of those who received the HL letter correctly answered questions about how common it is for women to have dense breasts (WA letter: 41.5% vs HL letter: 57.4%; *P* < .001) and the increased breast cancer risk (WA letter: 20.9% vs HL letter: 58.3%; *P* < .001). However, a significantly lower proportion of women correctly answered questions related to the masking effect (WA letter: 80.0% vs HL letter: 71.5%; *P* = .001) and density decrease with age (WA letter: 61.1% vs HL letter: 48.7%; *P* < .001).

Compared with the control group, significantly higher proportions of participants in the intervention groups reported perceiving their breast cancer risk their lifetime to be above average (control: 2.5%, WA letter: 8.9%, HL letter: 9.4%; *P* < .001). There was no significant difference between the 2 intervention groups (WA letter: 8.9% vs WA letter: 9.4%; *P* = .71).

## Discussion

Breast density notification legislation in the US has stimulated international discussion regarding whether and how best to include breast density notification alongside mammography results.^15,31,32^ This online randomized clinical trial was conducted in Australia where density notification is not routinely provided, with the exception of one state. The findings show that when screening participants are notified of their breast density via a letter accompanied by a brief informational pamphlet, their intention to seek supplemental screening and chances of feeling anxious, confused, and worried about developing breast cancer substantially increase. The findings also demonstrate that notified participants are more likely to intend to speak with a primary care practitioner. Providing breast density information in a format sensitive to lower HL did not make a substantial difference to the main outcomes of interest, except for some of the individual knowledge items. Ninety percent of the population in this study reported having adequate HL, and greater differences in outcomes using HL-sensitive information may be expected in populations with a higher prevalence of low HL.

Consistent with our findings, in a WA study that included a sample of women who were notified through a population-based screening program, 55% of women reported having consulted or were intending to speak with a primary care practitioner and 20% reported having an ultrasonography scan as an addition to their routine mammogram.^33^ Internationally, there are mixed opinions on breast density notification and any association with behavioral and psychological outcomes.^10^ A recent systematic review noted that, although most studies reported concerns, confusion, and a prevalence of anxiousness in approximately 40% of the participants who received notification, a small number of studies have not observed this association.^10^ As for screening intentions, the same review reported more consistent evidence across studies, pointing to higher intention to speak with a physician and seek additional screening in participants being notified and informed of their breast density.^10^ Previous studies have also shed light on the disparities in knowledge and awareness, as well as inequity, in accessing services in the context of notification.^15,34^

The long-term health outcomes of breast density notification at a population level remain unclear at this stage, insufficient to allow for detailed cost-effectiveness analysis. Based on limited smaller scale studies, the cost-effectiveness of supplemental screening depends on the modalities used, long-term outcomes used for calculating costs, and characteristics of the target group.^35,36^ A recent mathematical simulation study, based on findings of the DENSE study (a Dutch trial that assigned a group of women with extremely dense breasts to undergo magnetic resonance imaging screening), provides a new perspective.^37^ This simulation reported that taking into account the numbers of breast cancers, life-years, quality-adjusted life-years, breast cancer deaths, and overdiagnosis and associated health care costs, it can be cost-effective to offer women with extremely dense breasts magnetic resonance imaging screening at a 4-year interval.^37^ Other studies found mixed results, with one study concluding ultrasonography after a negative mammography is not cost-effective and the other reporting tomosynthesis plus mammography is cost-effective in women with dense breasts.^35,36^ Regardless, our study results can be useful for policy makers in discussion of potential costs associated with notification of breast density, with approximately 15% of women who are notified that they have dense breasts likely to seek supplemental screening, 25% likely to seek additional mammographic screening between routine schedules, and 65% likely to schedule a breast density–related primary care appointment. The widespread notification can also incur out-of-pocket costs for women who are not covered by publicly funded programs or privately insured, or when such services are not adequately covered by public or private health programs. This lack of coverage will have important implications for equity access to care.

Our work highlights that women are also likely to have heightened feelings of being anxious, confused, or worried about breast cancer risk after being notified that they have dense breasts. An important next step before rolling out widespread notification is to conduct a randomized clinical trial with women who are notified through population-based screening programs in real-life settings to test these outcomes and set up processes to longitudinally evaluate outcomes both quantitatively and qualitatively. This information can assist planning to ensure relevant services are available if density notification is broadly introduced, including additional information and support for both the women and GPs, and a clinical management pathway to progress to supplemental screening. In doing so, the increased rates of false-positive findings with supplemental screening also need to be taken into consideration.^38^

Previous studies have shown that GPs in Australia and primary care practitioners in the US had limited knowledge about breast density, were uncertain about how to manage care for women with dense breasts, and needed support and training.^39,40^ With an expected increase in GP consultations related to breast density and cancer risk if notification is introduced, it will be crucial to ensure GPs have the knowledge and resources to counsel individuals about breast density and the pros and cons of supplemental screening, including in relation to psychological well-being. Informed and shared decision-making models based on evidence, patient values and goals, and joint decisions may help to reduce worries and other adverse psychological outcomes.^41,42^

Outside patient-clinician consultations, written or other materials providing evidence-based and unbiased information available through easily accessible channels can facilitate informed health care decisions.^43^ In this study, participants in both intervention groups were significantly more likely to have accurate knowledge about breast density than those in the control group. However, we did not find an association between HL-sensitive information and the main outcome measures, such as screening intentions and psychological effects. Although this finding may be explained by the high level of HL among our sample, it also demonstrates an interest in supplemental screening related to breast density information regardless of additional information on both the potential benefits and harms of supplemental screening. Further research in this area that includes samples of people with low HL not recruited online is needed. Previous studies have noted that the degree to which HL interventions may have an influence varies depending on the HL level of the target audience, and the lower the HL level, the larger the possible influence.^44^ Regardless, at this time HL-tailored information may still be useful in the wider community if breast density notification is introduced, and future research on what format of information achieves the best outcomes is warranted.

### Strengths and Limitations

The key strength of this study is the randomization of interventions with participants having similar baseline characteristics across groups. This trial was conducted among screening-aged Australian residents who live in areas with no widespread breast density notification and therefore are unlikely to be biased by prior familiarity with breast density information. To our knowledge, this is the first study to examine the effect of HL-sensitive breast density information on key relevant outcomes.

The study has limitations. Despite oversampling for low educational level during the recruitment process and ensuring that 40% of participants had lower educational attainment in line with the national level,^20^ there was a high proportion of participants with adequate HL (>90%). This increased level may be explained by the association between higher HL and interest in research participation^45^ and the online nature of this study. Because this study was hypothetical (since breast density notification is not endorsed in the national screening program in Australia), women who receive a dense breast notification in real-life settings may respond differently than the participants in our study. Online studies using hypothetical clinical scenarios have been widely used in both psychology and health fields for many years and have been reported to have clinically meaningful findings.^46^ Randomized clinical trials comparing women with dense breasts who are notified with those who are not notified through population-based screening programs are needed to validate or refute these findings. To minimize the effect of this limitation, our standardized screening mammogram results letters were adapted from currently used letters. The letters, however, may not be suited or directly relevant to other international screening settings owing to differences in health systems, screening policies, and programs.

## Conclusions

In countries outside the US without mandated breast density notification, more evidence on overall benefits and harms of notification, as well as adequate and equitable service planning and communication strategies, are needed to inform future screening policy decisions about density notification. Although research from the US since the legislation of breast density notification provides invaluable insight into what to expect after introducing a mandated notification, other countries should be able to generate local evidence before widespread density notification. The findings of this trial, such as increased demand for services and adverse psychological outcomes associated with notification, have important implications for policy makers in Australia and countries with breast screening programs in considering the outcomes of potential widespread notification of breast density.

## Notes

Supplement 1.
Trial Protocol and Statistical Analysis Plan

Supplement 2. **eAppendix.** Online Questionnaire Including Control and Intervention Materials
eReferences
**eTable.** Change in Cancer Worry

Supplement 3.
Data Sharing Statement
