# Supplementary material for: Australian Women’s Intentions and Psychological Outcomes Related to Breast Density Notification and Information: A Randomized Clinical Trial
Source: JAMA Netw Open. 2022 Jun 16;5(6):e2216784. doi: 10.1001/jamanetworkopen.2022.16784 (PMC9204548; doi:10.1001/jamanetworkopen.2022.16784)
Supplement: Supplement 1. — Trial Protocol and Statistical Analysis Plan [file jamanetwopen-e2216784-s001.pdf]

Supplement 1. Trial protocol and statistical analysis plan

Trial registered on ANZCTR

**Registration number**	ACTRN12621000253808
**Ethics application status**	Approved
**Date submitted**	1/01/2021
**Date registered**	9/03/2021
**Date last updated**	10/11/2021
**Date data sharing statement initially provided**	9/03/2021
**Date results information initiallyprovided**	10/11/2021
**Type of registration**	Prospectively registered

Titles & IDs

**Public title**	Examination of Australian women's intentions and psychological outcomes related to breast densitynotification with mammography screening results: An online randomised experiment
**Scientific title**	Examination of Australian women's intentions and psychological outcomes related to breast densitynotification with mammography screening results: An online randomised experiment
**Secondary ID [1]**	Nil known
**Universal Trial Number (UTN)**	
**Trial acronym**	
**Linked study record**	

Health condition

Health condition(s) or problem(s) studied:

Breast density

Breast cancer screening

Breast cancer

**Condition category**	**Condition code**
Public Health	Other public health
Cancer	Breast

Intervention/exposure

**Study type**	Interventional
**Description of intervention(s) / exposure**	This study aims to quantitively assess how different formats of information provision on breast density affect women's screening intentions, psychological outcomes (anxiety and breast cancer worry) and knowledge of breast density.<br><br>This study will use a 3-arm randomised trial design. This trial will be conducted online through a survey platform. An online sample of Australian women (outside of Western Australia) will be recruited through a large market research organisation. Women will be aged between 40 to 74 years with no prior history of breast cancer or ductal carcinoma in situ (DCIS). Women will not be required to have undergone screening mammogram prior to participating in this study.<br><br>Women will be presented with a hypothetical scenario in which they were asked to imagine going for a routine mammogram screening and receiving a letter about their mammogram results. Women then will be randomised to be shown one of three example letters (written format) about mammography results.<br><br>Arm 1: Control group (standard screening mammogram result)<br>Women in the control group will be shown a standard screening mammogram result letter, which will show a "clear" result, i.e., no breast cancer could be seen on the mammogram. This letter will be similar to the ones that breast screening services in Australia send out to women with 'clear' results.<br><br>Arm 2: Intervention 1 (standard screening mammogram result + existing breast density notification messaging (e.g. WA))<br>Women in the intervention 1 will be shown the same letter as the control group, along with an additional breast density notification letter for women with dense breasts. This breast density letter will be identical to the ones that screening services in Western Australia send out to women who are found to have dense breast tissue. This letter includes information that the woman's breast tissue is dense, followed by information on what breast density means, its implications and following up with a GP.<br><br>Arm 3: Intervention 2 (standard screening mammogram + health literacy sensitive breast density messaging)<br>Women in the intervention 2 will be shown the same letter as the control group, along with an additional breast density notification letter for women with dense breasts. This letter will be developed by the research team and will be health literacy appropriate, i.e. tailored for a reading level of grade 8. It will also incorporate health literacy strategies such as avoiding complex medical terminologies, using spaces and images or icons. This letter will include information such as woman's breast density level, what is breast density and its implications, what can be done, what are the pros and cons of additional testing and speaking with a healthcare provider.<br><br>The online intervention will be administered once only with no repeat or subsequent sessions upon completion. It will take 10-15 minutes for participants to take part in the study.<br><br>Adherence to the intervention will be ensured by forcing a response for outcome measurement items that follow the intervention and by monitoring the overall time taken to complete the online study.
**Intervention code [1]**	Behaviour
**Comparator / control treatment**	The control group will be randomised to receive a screening mammogram result without the breast density messaging.
**Control group**	Active

Outcomes

**Primary outcome [1]**	Screening intention<br><br>This outcome is a composite outcome comprising of four elements: intention to go for extra tests, choice of extra tests, intention to change screening frequency and intended change in screening frequency (increase or decrease).<br><br>Participants will be asked if they would go for extra tests (supplemental screening) after receiving the intervention or control messaging (Yes/No/Don't know). If they choose yes, they will be further prompted to choose which extra test (ultrasound, MRI, ultrasound and MRI, tomosynthesis, or other) they would go for. Participants will be asked whether they would change how often they go for screening mammogram after receiving the intervention or control messaging (Yes/No/Don't know) and if yes, whether they would go more frequently or less frequently.
Timepoint [1]	Immediately after intervention
**Primary outcome [2]**	Anxiety<br><br>The following item will be used to measure anxiety.<br><br>Please indicate the extent of your agreement or disagreement with the following statements. Receiving this letter does/would make me feel anxious (uneasy, worried, nervous)*<br>4-point scale (1 = ‘strongly agree’ to 4 = ‘strongly disagree’)

Reference: Rhodes DJ, Jenkins SM, Hruska CB, et al. Breast Density Awareness, Knowledge, and Attitudes Among US Women: National Survey Results Across 5 Years. J Am Coll Radiol 2020;17(3):391-404. doi: 10.1016/j.jacr.2019.11.003 [published Online First: 2019/11/23]

Timepoint [2]

Immediately after intervention

Primary outcome [3]

Breast cancer worry

The following item will be used to measure breast cancer worry.

How worried would you be about developing breast cancer after receiving this letter? 4-point scale (1 = ‘strongly agree’ to 4 = ‘strongly disagree’)

Reference: Sutton S, Bickler G, Sancho-Aldridge J, Saidi G. Prospective study of predictors of attendance for breast screening in inner London. Journal of Epidemiology & Community Health. 1994;48(1):65-73.

Timepoint [3]

Immediately after intervention

Secondary outcome [1]

Breast density-related knowledge

This outcome is a composite outcome comprising of four elements: knowledge of the prevalence of women with dense breasts, knowledge that breast density is an independent risk factor of breast cancer, knowledge of dense breast tissue's masking effect on a mammogram and knowledge of breast density change with age.

Breast density-related knowledge will be measured using both the custom-designed and previously validated items. The items will measure participants' knowledge in relation to the prevalence of women with dense breasts, breast density being an independent risk factor of breast cancer, breast density masking effect on a mammogram and change with age.

Reference: Rhodes DJ, Jenkins SM, Hruska CB, et al. Breast Density Awareness, Knowledge, and Attitudes Among US Women: National Survey Results Across 5 Years. J Am Coll Radiol 2020;17(3):391-404.

Timepoint [1]

Immediately after intervention

Secondary outcome [2]

Intention to talk to a doctor about breast density will be measured using a single item.

Would you plan to talk to your doctor or nurse about this letter? Yes/No/Don't know

Timepoint [2]

Immediately after intervention

Secondary outcome [3]

Breast cancer perceived risk will be measured using the following items:

What do you think is your chance of getting breast cancer in your lifetime if you received this letter? Below average/Average/Above average

Compared to other people your age, race and sex, what do you think is your chance of getting breast cancer in your lifetime if you received this letter?
Below average/Average/Above average

Reference: Lipkus IM, Kuchibhatla M, McBride CM, Bosworth HB, Pollak KI, Siegler IC, et al. Relationships among Breast Cancer Perceived Absolute Risk, Comparative Risk, and Worries. Cancer Epidemiology Biomarkers & Prevention. 2000;9(9):973-975.

Timepoint [3]

Immediately after the intervention

Secondary outcome [4]

Other psychological impacts of breast density messaging will be measured using the

following items: The following item will be used to measure anxiety.

Please indicate the extent of your agreement or disagreement with the following statements. Receiving this letter does/would make me feel informed to make decisions regarding my breast health 4-point scale (1 = ‘strongly agree’ to 4 = ‘strongly disagree’)

	Receiving this letter does/would make me feel confused about what to do regarding my breast health 4-point scale (1 = ‘strongly agree’ to 4 = ‘strongly disagree’)<br><br>Reference: Rhodes DJ, Jenkins SM, Hruska CB, et al. Breast Density Awareness, Knowledge, and Attitudes Among US Women: National Survey Results Across 5 Years. J Am Coll Radiol 2020;17(3):391-404. doi: 10.1016/j.jacr.2019.11.003 [published Online First: 2019/11/23]
Timepoint [4]	Immediately after the intervention

Eligibility

**Key inclusion criteria**	Australian women aged 40-74, live outside Western Australia and no prior history of breast cancer or DCIS. Women will not be required to have undergone a screening mammogram prior to participation in the study.
**Minimum age**	40 Years

Maximum age 74 Years

Gender Females

Can healthy volunteers participate? Yes

Key exclusion criteria Male, aged under 40 or over 74, lives in Western Australia, has a prior history of breast cancer or DCIS

Study design

Purpose of the study Educational / counselling / training

Allocation to intervention Randomised controlled trial

Procedure for enrolling a subject and allocating the treatment (allocation concealment procedures) Central randomisation by computer using Qualtrics survey software.

Researchers will have no direct contact with participants. Once a participant agrees to take part in the study they will be randomly assigned to be immediately sent a questionnaire containing either one of two versions of the intervention or the control.

Methods used to generate the sequence in which subjects will be randomised (sequence generation) Qualtrics computer software will randomly allocate each participant to one of the arms of the trial as they enter the study.

Masking / blinding Blinded (masking used)

Who is / are masked / blinded? The people receiving the treatment/s
The people administering the treatment/s

Intervention assignment Parallel

Other design features

Phase Not Applicable

Type of endpoint(s) Efficacy

Statistical methods / analysis The proposed sample (n=1398) provides 80% with an adjusted alpha of 0.025 to account for multiple comparisons between either intervention arm and the control. Statistical analysis will be conducted using planned contrasts between intervention arms and control and implemented in regression models. All data will be analysed by researchers at the University of Sydney and compiled for publication and dissemination.

Recruitment

Recruitment status Completed

Date of first participant enrolment

Anticipated 1/05/2021 **Actual** 4/07/2021

Date of last participant enrolment

Anticipated 1/06/2021 **Actual** 30/08/2021

Date of last data collection

Anticipated 1/06/2021 **Actual** 30/08/2021

Sample size

Target 1398 **Accrual to date** **Final** 1505

Recruitment in Australia

Recruitment state(s) ACT,NSW,NT,QLD,SA,TAS,VIC

Ethics approval

**Ethics application status**	Approved
**Ethics committee name [1]**	University of Sydney Human Research Ethics Committee
**Ethics committee address [1]**	Research Integrity and Ethics Administration | Research Portfolio<br>Level 3, Administration Building (F23) | The University of Sydney | NSW | 2006
**Ethics committee country [1]**	Australia
**Date submitted for ethics approval [1]**	16/11/2020
**Approval date [1]**	24/02/2021
**Ethics approval number [1]**	2020/858

Summary

Brief summary

This study is investigating how different formats of information provision on breast density affect women's screening intentions, psychological outcomes and knowledge of breast density.

Who is it for?
You may be eligible for this trial if you are female, aged 40-74 years, live outside Western Australia and have no prior history of breast cancer or ductal carcinoma in situ.

Study details
Participants in this study will complete an online questionnaire and during which will be presented with a hypothetical scenario of undergoing a routine screening mammogram and receiving the results.
Participants will be randomised to receive control information (screening mammogram result without the breast density messaging) or one of two versions of the intervention with breast density information via notification messaging. Participants will then be asked to complete questions in response to the information they have received.

It is hoped this study will inform researchers of how different formats of information on breast density impact women's screening intentions and psychological outcomes.

Trial website

Trial related presentations / publications

Public notes

Contacts

Principal investigator

**Name**	Dr Brooke Nickel
**Address**	Room 127A, Edward Ford Building A27 | The University of Sydney | NSW | 2006
**Country**	Australia
**Phone**	+61 2 9351 7829
Fax	
**Email**	brooke.nickel@sydney.edu.au

Contact person for public queries

**Name**	Dr Hankiz Dolan
**Address**	Room 128C, Edward Ford<br>Building (A27) The University of<br>Sydney NSW 2006

**Country**	Australia
**Phone**	+61 2 9351 7789
Fax	
**Email**	hankiz.dolan@sydney.edu.au

Contact person for scientific queries

**Name**	Dr Hankiz Dolan
**Address**	Room 128C, Edward Ford<br>Building (A27)The University of<br>Sydney<br>NSW 2006
**Country**	Australia
**Phone**	+61 2 9351 7789
Fax	
**Email**	hankiz.dolan@sydney.edu.au

Data sharing statement

**Will individual participant data(IPD) for this trial be available (including data dictionaries)?**	No
No/undecided IPD sharing reason/comment	In line with our ethics approval, participant data will be made anonymous and will be aggregated for thepurpose of statistical analyses. Findings will be reported at the group/condition level rather than the individual participant level.<br>No other documents available
**What supporting documents are/will be available?**	No

Summary results

Have study results been published in a peer-reviewed journal?

Other publications

Have study results been made publicly available in another format?

Statistical analysis plan

Working title: Australian women's intentions and psychological outcomes related to breast density notification and information with mammography screening results: An online randomized experiment

Broad aims: This study aims to quantitively assess how information provision of breast density information affects women’s intention to seek supplemental screening and self-reported psychological outcomes.

Outcome measures:

Variable name in SPSS in light blue

Variable for comparison: condition (condition)

Primary outcomes:

- intentions to seek supplemental screening (Intent_Screen_SuppScreen)
- psychological outcomes
  - feeling anxious (Anxiety)
  - feeling informed (Informed)
  - feeling confused (Confusion)
- breast cancer worry (CancWorryAfter)

Secondary outcomes:

- intention to speak to a GP (Intent_TalkGP)
- breast density knowledge
  - Knowledge about percentage (BDKnowl_Percentage)
  - Knowledge about cancer risk (BDKnowl_CancRisk)
  - Knowledge about masking (BDKnowl_Masking)
  - Knowledge about age (BDKnowl_Age)
- Cancer risk perception (Percep_CancRisk)

Specific research questions:

1. Do outcomes relating to intention to seek supplemental screening, feelings of being anxious, informed or confused, breast cancer worry, breast density knowledge, cancer riskperception and intention to speak to a GP differ between women who received screening mammography results with or without breast density messaging?
2. Do outcomes relating to intention to seek supplemental screening, feelings of being anxious, informed or confused, breast cancer worry, breast density knowledge, cancer riskperception and intention to speak to a GP differ between women who received existing breast density notification messaging and health literacy sensitive breast density messaging?
3. Do women’s breast cancer worry differ before and after the breast density notification? Does the difference vary depending on which messaging they receive?
   - Intervention (condition=2 or condition=3)
   - Cancer worry before intervention (CancWorryBefore)
   - Cancer worry after intervention (CancWorryAfter)
