# Supplementary material for: Australian Women’s Intentions and Psychological Outcomes Related to Breast Density Notification and Information: A Randomized Clinical Trial
Source: JAMA Netw Open. 2022 Jun 16;5(6):e2216784. doi: 10.1001/jamanetworkopen.2022.16784 (PMC9204548; doi:10.1001/jamanetworkopen.2022.16784)
Supplement: Supplement 2. — eAppendix. Online Questionnaire Including Control and Intervention Materials eReferences eTable. Change in Cancer Worry [file jamanetwopen-e2216784-s002.pdf]

Supplementary Online Content

Dolan H, McCaffery K, Houssami N, et al. Australian women's intentions and psychological outcomes related to breast density notification and information: a randomized clinical trial. *JAMA Netw Open*. 2022;5(6):e2216784. doi:10.1001/jamanetworkopen.2022.16784

eAppendix. Online Questionnaire Including Control and Intervention Materials

eReferences

eTable. Change in Cancer Worry

This supplementary material has been provided by the authors to give readers additional information about their work.

© 2022 Dolan H et al. *JAMA Network Open.*

eMethod: Online Questionnaire Including Control and Intervention Materials

Australian women's intentions and psychological outcomes related to breast density notification: a randomised experiment

[Survey Landing Page]

Thank you for your interest in our study about breast density.

Please take the time to read through the Participant Information Statement and Consent Form using the links below.

[Attach Participant Information Statement and Consent Form]

This study is completely voluntary and you do not have to take part.

If you are interested in taking part in this study, you will be asked to consent to take part by ticking the 'yes' box at the beginning of the questionnaire. By giving your consent to take part in this study, you are telling us that you:

- ✓ Understand what you have read in the Participant Information Statement and Consent Form.
- ✓ Agree to take part in the research study as outlined in Participant Information Statement and Consent Form.
- ✓ Agree that your participation is anonymous
- ✓ Agree that the information you enter into the questionnaire will be used for the research study

When you have consented, you will fill out a questionnaire that asks some questions, such as:

- Demographic questions, such as age, education, income level and relationship status
- General health and cancer related questions
- Breast cancer knowledge and breast screening history related questions.
- You will be randomized to read one of three HYPOTHETICAL EXAMPLES (these are **made up** examples) in which you receive letters about mammography results. **Please note that you WILL NOT be receiving information or advice on your real mammogram result or breast density.**
- The hypothetical examples will be followed by questions about breast density, screening intentions, anxiety and cancer worry.

You can withdraw your responses any time before you have submitted the questionnaire. Once you have submitted it, your responses cannot be withdrawn because they are anonymous.

If you would like to know more at any stage during the study, please feel free to contact Dr Hankiz Dolan (hankiz.dolan@sydney.edu.au) or Dr Brooke Nickel (brooke.nickel@sydney.edu.au)

The ethical aspects of this study have been approved by the human research ethics committee of the University of Sydney [Ref: 2020/858] (Telephone: +61 2 8627 8176; Email: human.ethics@sydney.edu.au)

© 2022 Dolan H et al. *JAMA Network Open.*

Screening questions

Before we get started, we would like to confirm a few details with you.

1. Which of the following best describes your current gender identity?
    a. Female
    b. Male
    c. Non-binary/gender fluid
    d. Different identity

[b,c,d terminate survey; display end of survey message]

2. What is your age?
    a. 18-24
    b. 25-29
    c. 30-34
    d. 35-39
    e. 40-44
    f. 45-49
    g. 50-54
    h. 55-59
    i. 60-64
    j. 65-69
    k. 70-74
    l. 75 and over

[a, b, c, d, I terminate survey, display end of survey message]

3. Which Australian state or territory do you currently live in?
    a. New South Wales
    b. Victoria
    c. Australian Capital Territory
    d. Queensland
    e. South Australia
    f. Western Australia
    g. Northern Territory
    h. Tasmania

[f terminates survey, displays end of survey message]

4. Have you been previously diagnosed with breast cancer?
    a. Yes
    b. No
5. Have you been previously diagnosed with ductal carcinoma in situ (DCIS)?
    a. Yes
    b. No

[a terminates survey, displays end of survey message]

CONSENT

Do you consent to take part in this study as described in the Participant Information Statement and Consent Form?

a. Yes
b. No [Terminate the survey]

End of survey message: Thanks again for your interest in taking part in our study. Unfortunately, you do not meet our eligibility criteria for participating.

© 2022 Dolan H et al. *JAMA Network Open.*

QUESTIONNAIRE FOR WOMEN

SECTION 1: SOCIO-DEMOGRAPHIC

In this section, we would like to ask you a few demographic questions:

2. What is your postcode where you currently live? (suburb name if not sure)
[free text]

3. What is your highest level of education? Please tick below.
    a. Postgraduate degree (Master's or Doctorate)
    b. Graduate diploma/Graduate certificate
    c. Bachelor's degree
    d. Advanced diploma/diploma
    e. Certificate III/IV
    f. Certificate I/II
    g. Year 12
    h. Year 11
    i. Year 10 or below
    j. Level not determined
    k. Don't know

4. What is your current employment status? Please tick below.
    a. Permanent or ongoing
    b. Casual/temporary (no paid sick leave or annual leave)
    c. Fixed-term contract
    d. Self-employed
    e. On paid leave (e.g. maternity leave)
    f. Unemployed
    g. Not working/not in the labour force (e.g., student, home-duties, retired)

5. What is your total household income in 2021 (before taxes)?
    a. Less than $25000
    b. Between $25,000-$50,000
    c. Between $50,000-$75,000
    d. Between $75,000-$100,000
    e. Between $100,000-$125,000
    f. More than $125,000

6. What is your current relationship status? Please tick below.
    a. Married or de-facto partnership
    b. In a relationship but not living together
    c. Single, never married
    d. Widowed
    e. Divorced or separated

7. How many children do you have?
    a. None
    b. 1
    c. 2-4
    d. More than 4
    e. Prefer not to say

8. Are you of Aboriginal and/or Torres Strait Islander origin?
    a. Aboriginal origin
    b. Torres Strait Islander origin
    c. Both Aboriginal and Torres Strait Islander origin
    d. Neither

© 2022 Dolan H et al. *JAMA Network Open.*

9. Were you born in Australia?
    a. Yes
    b. No

    [IF NO]
    In which country were you born? Please specify_________________
    In what year did you move to Australia to live?________________

10. What is the main language you speak at home? Please tick below.
    a. English
    b. Other: please specify _______________________________________

11. Do you have private health insurance? Please tick below.
    a. Yes
    b. No
    c. Don't know

SECTION 2: GENERAL HEALTH

12. In general, would you say your health is…[1^]
    a. Excellent
    b. Very good
    c. Good
    d. Fair
    e. Poor

13. Have you ever been diagnosed with cancer? Please tick below.
    a. Yes
    b. No
    c. Don't know

    [IF YES]
    14. which type of cancer? Please tick below.
        a. Bowel
        b. Cervical
        c. Lung
        d. Lymphoma
        e. Melanoma
        f. Prostate
        g. Thyroid
        h. Other: ____________________________
        i. Don't know

14. Has anyone in your immediate family (parents, siblings or children) ever been diagnosed with cancer? Please tick below. [2]
    a. Yes
    b. No
    c. Don't know

    [IF YES]
    15. Which type of cancer? Please tick all that apply.
        a. Bowel
        b. Breast
        c. Cervical
        d. Lung
        e. Lymphoma
        f. Melanoma
        g. Prostate
        h. Thyroid
        i. Other: __________________________

© 2022 Dolan H et al. *JAMA Network Open.*

j. Don't know

[**IF BREAST**]

16. Who was this?[2] (Please tick all that apply)
    a. Mother
    b. Grandmother
    c. Aunt
    d. Sister
    e. Daughter
    f. Niece
    g. **[TEXT BOX]** Other, please specify:

17. How worried are you about developing breast cancer? Please tick below.[3]
    a. Not worried at all
    b. A bit worried
    c. Quite worried
    d. Very worried

18. The following questions are related **how you have been feeling over the past two weeks**. Please read each statement and then choose the most appropriate option regarding how you felt in the **last two weeks**.[4^]

	All of the time	Most of the time	More than half of the time	Less than half of the time	Some of the time	At no time
I have felt cheerful in good spirits						
I have felt calm and relaxed						
I have felt active and vigorous						
I woke up feeling fresh and rested						
My daily life has been filled with things that interest me.						

SECTION 3: HEALTH LITERACY

19. How often do you need to have someone help you when you read instructions, pamphlets or other written material from your doctor or pharmacy?[5^]
    a. Always
    b. Often
    c. Sometimes
    d. Occasionally
    e. Never

© 2022 Dolan H et al. *JAMA Network Open.*

Participants will be randomised to view one of the following three letters about mammography results with or without the breast density notification/messaging

Study Arm 1 (Control)

Please read the information below and answer the questions that follow. Please note that you will be asked to imagine as if the following information is true. Please answer how you would feel or react if you were in this situation, to the best of your ability.

Please imagine that you have gone to a routine mammogram screening for breast cancer and received the following letter about your mammogram results.

[Timer for reading: 30 seconds]

© 2022 Dolan H et al. *JAMA Network Open.*

Your mammogram (breast x-ray) result:
No breast cancer could be seen on your mammogram

Your recent screening mammogram showed no evidence of breast cancer.

When should you next visit BreastScreen?
You are eligible for a mammogram every two years through BreastScreen.

Mammography can detect 70 - 90% of breast cancers. There is a small chance that an existing breast cancer may not be seen on a mammogram.

New Breast cancers can develop between screening visits. If you notice any changes in your breasts, such as breast lumps, nipple discharges, changes in the breast shape or in the skin of the breast, or new and persistent breast pain, you should contact your GP immediately.

BreastScreen sends reminder letter to women aged 50 to 74 years. However, all women over the age of 40 can access screening with BreastScreen by calling 132050 to arrange an appointment.

Thank you for attending BreastScreen.

Yours sincerely,

Study Arm 2 (Intervention one)

Please read the information below and answer the questions that follow. Please note that you will be asked to imagine as if the following information is true. Please answer how you would feel or react if you were in this situation, to the best of your ability.

Please imagine that you have gone to a routine mammogram screening for breast cancer and received the following letter about your mammogram results.
[Timer for reading: 30 seconds]

© 2022 Dolan H et al. *JAMA Network Open.*

Your mammogram (breast x-ray) result:
No breast cancer could be seen on your mammogram

Your recent screening mammogram showed no evidence of breast cancer.

When should you next visit BreastScreen?
You are eligible for a mammogram every two years through BreastScreen.

Mammography can detect 70 - 90% of breast cancers. There is a small chance that an existing breast cancer may not be seen on a mammogram.

The screening mammogram showed that your breasts are dense. Although mammograms are less sensitive in finding breast cancer in women with dense breasts, current evidence shows that screening mammograms are still the best test for breast cancer detection. Please speak to your GP about your breast density and what it means for you. More information about dense breasts are given on the next page.

New breast cancers can develop between screening visits. If you notice any changes in your breasts, such as breast lumps, nipple discharges, changes in the breast shape or in the skin of the breast, or new and persistent breast pain, you should contact your GP immediately.

BreastScreen sends reminder letter to women aged 50 to 74 years. However, all women over the age of 40 can access screening with BreastScreen by calling 132050 to arrange an appointment.

Thank you for attending BreastScreen.

Yours sincerely,

[Timer for reading: 40 seconds]

© 2022 Dolan H et al. *JAMA Network Open.*

Information for Women
Dense Breasts

Your breast X-rays show that your breast tissue is dense. This is normal. Breasts are usually denser in younger women. They naturally become less dense as the breasts change with increasing age and the glandular tissue decreases.

When breasts are dense on an X-ray it is more difficult to see abnormal changes, including the early signs of breast cancer. Therefore, it is important for you to have a careful breast examination by your doctor as well as the breast X-rays.

If you have nominated a General Practitioner for result notifications, she/he will receive a similar letter and will be expecting to see you. Please contact your GP to make an appointment to have a breast check.

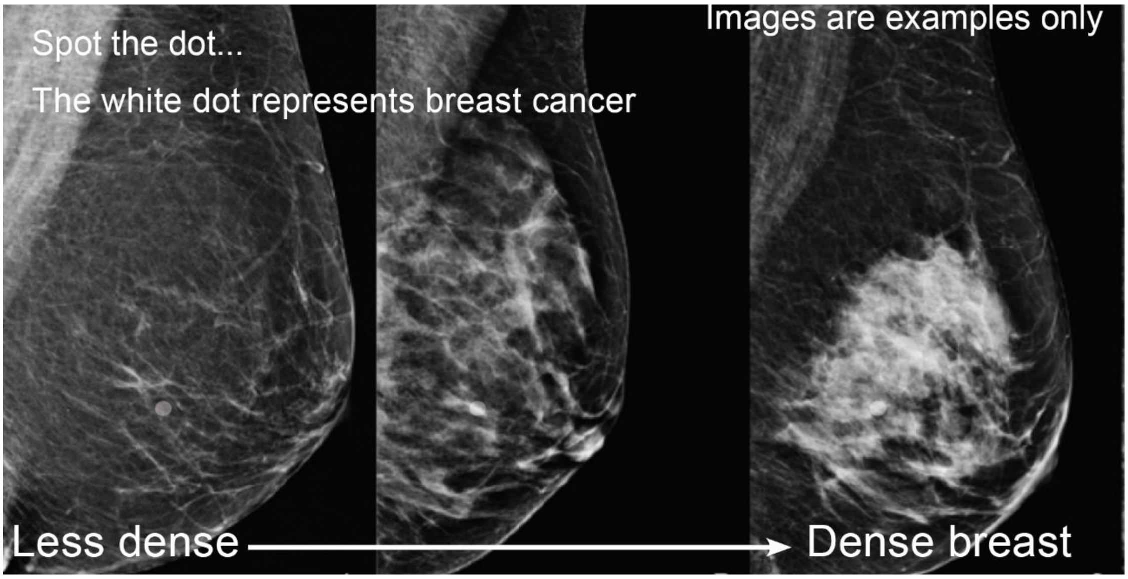

The greater the density, the more white areas on the breast X-ray, and the less obvious the dot.

Be breast aware

Get to know your breasts and what is normal for you. Look in the mirror at your breasts and feel your breasts from time to time.

If you notice any unusual changes in your breasts such as lumps, nipple discharge, or persistent new breast pain, even if your last screening mammogram was normal, please see your GP promptly.

© 2022 Dolan H et al. *JAMA Network Open.*

Study Arm 3 (Intervention two)

Please read the information below and answer the questions that follow. Please note that you will be asked to imagine as if the following information is true. Please answer how you would feel or react if you were in this situation, to the best of your ability.

Please imagine that you have gone to a routine mammogram screening for breast cancer and received the following letter about your mammogram results.

[Timer for reading: 30 seconds]

Your mammogram (breast x-ray) result:
No breast cancer could be seen on your mammogram

Your recent screening mammogram showed no evidence of breast cancer.

When should you next visit BreastScreen?
You are eligible for a mammogram every two years through BreastScreen.

Mammography can detect 70 - 90% of breast cancers. There is a small chance that an existing breast cancer may not be seen on a mammogram.

The screening mammogram showed that your breasts are dense. Although mammograms are less sensitive in finding breast cancer in women with dense breasts, current evidence shows that screening mammograms are still the best test for breast cancer detection. Please speak to your GP about your breast density and what it means for you. More information about dense breasts are given on the next page.

New breast cancers can develop between screening visits. If you notice any changes in your breasts, such as breast lumps, nipple discharges, changes in the breast shape or in the skin of the breast, or new and persistent breast pain, you should contact your GP immediately.

BreastScreen sends reminder letter to women aged 50 to 74 years. However, all women over the age of 40 can access screening with BreastScreen by calling 132050 to arrange an appointment.

Thank you for attending BreastScreen.

Yours sincerely,

[Timer for reading: 60 seconds]

© 2022 Dolan H et al. *JAMA Network Open.*

What is breast density?

Breasts are made up of three types of tissue:

- fatty tissue
- fibrous tissue
- glandular tissue

Breasts are considered dense if they have:

- More fibrous and glandular tissue
- Less fatty tissue

There are four categories of breast density.

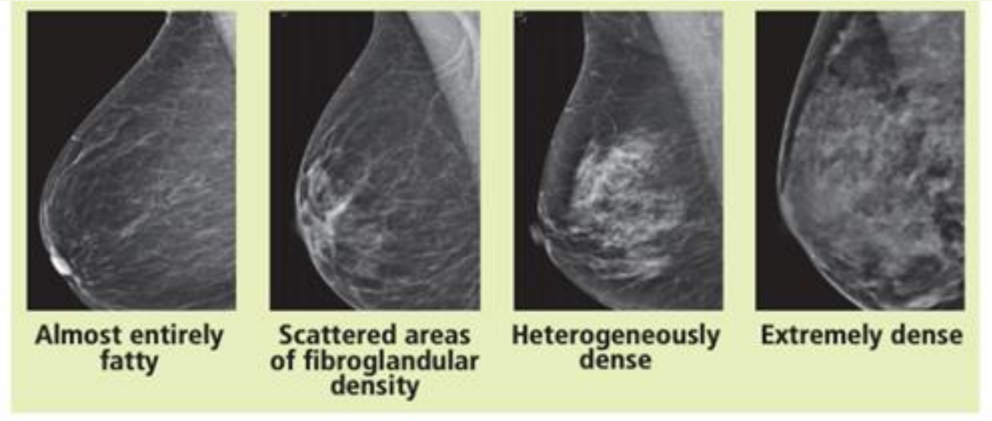

Image source: American College of Radiology https://www.acr.org/-/media/ACR/Files/Breast-Imaging-Resources/Breast-Density-bro_ACR_SBI.pdf

Breast density can only be seen on mammogram.

Usually, a radiologist reads your mammogram image. They decide which category best describes your breast density level. Sometimes they might use a computer software.

Breast density is not about how breasts feel, look, the size of them and the firmness.

The breasts usually get <u>less dense</u> as women get older.

Why does breast density matter?

There are many factors, such as age, family history, genetics, being overweight and inactive lifestyle, that can increase your risk of developing breast cancer in your lifetime.

<u>Having dense breasts</u> can increase your risk of developing breast cancer. The increased risk is smaller compared other risk factors, such as family history and genetics.

© 2022 Dolan H et al. *JAMA Network Open.*

Dense breast tissue might make it harder to spot a lump/tumor on a mammogram image.

How common is it to have dense breasts?

The estimates vary.

It depends on a few factors, including the age of the screening population. It also depends on how breast density is measured and classified.

For example, if more younger women than older women are having a screening mammogram in a population, the proportion of women with dense breasts will be higher.

Studies from the United States and other countries estimate that, around 25% to 50% women over the age of 40 are likely to have dense breasts.

What can I do if mammogram shows my breasts are dense?

Please speak to your GP about your breast density and what it means for you.

Be alert, not alarmed

- ❖ It's common for women to have dense breasts.
- ❖ Just like your age and family history, you cannot modify your breast density.
- ❖ Supplemental screening (breast imaging additional to mammogram) by breast ultrasound or MRI, or more frequent mammograms, might improve the detection of cancer in dense breasts.
- ❖ However, supplemental screening increases the chances of:
    - ➢ **False-positive results:** suggesting a cancer is present, when in fact there is no cancer
    - ➢ **Overdiagnosis:** being diagnosed with a condition that will never cause symptoms or death during your remaining lifetime
    - ➢ **Overtreatment:** being treated for a condition that would never have caused you symptoms or death
    - ➢ Distress, fear, anxiety, treatment side-effects, and reduced quality of life

❖ **Mammogram is the only screening method that is shown to reduce breast cancer deaths at a population level.**

❖ Always stay vigilant to changes in your breasts. Speak to your GP if you have any concerns.

© 2022 Dolan H et al. *JAMA Network Open.*

SECTION 4: OUTCOME MEASURES

24. After receiving this letter, would you plan to talk to your GP about it? *
    a. Yes
    b. No
    c. Don’t know

25. What of the following options would you want to do after receiving this letter?
    a. Nothing different, I would go to my next routine mammography in 2 years
    b. Go for breast screening more often, i.e., once a year
    c. Go for breast screening less often
    d. Seek supplemental screening or MRI
    e. Don’t know

26. Would you be willing to pay for supplemental screening out of pocket? *
    a. Yes
    b. No
    c. Don't know

28. Please indicate the extent of your agreement or disagreement with the following statements. Receiving this letter would make me feel:[2]

	Strongly agree	Agree	Disagree	Strongly disagree
Anxious (uneasy, worried, nervous)				
Informed to make decisions regarding my breast health				
Confused about what to do regarding my breast health				

29. How worried would you be about developing breast cancer after receiving this letter?[3]
    a. Not worried at all
    b. A bit worried
    c. Quite worried
    d. Very worried

30. Compared to other women your age, race and sex, what do you think is your chance of getting breast cancer in your lifetime if you received this letter?[6]
    a. Below average
    b. Average
    c. Above average

The following questions are related to breast density. Please select an answer that best reflects what you know about breast density. If you don’t know, or feel unsure, or have never read or heard of it, please choose the answer ‘Don’t know’.

20. Approximately, what percentage of women of breast screening age (40-74 years) have dense breasts?*
    a. <25%
    b. 25%-50%
    c. 50%-75%
    d. >75%
    e. Don’t know

© 2022 Dolan H et al. *JAMA Network Open.*

21. Having dense breasts can increase your risk of breast cancer.*
    a. True
    b. False
    c. Don't know

22. If a woman has dense breasts, what impact does this have on the ability of a mammogram to correctly detect cancer?[2]
    a. Dense breasts make it easier to see cancer on a mammogram
    b. Dense breasts do not impact the ability to see cancer on a mammogram
    c. Dense breasts make it more difficult to see cancer on a mammogram
    d. Don't know

23. Does a woman's breast density change with age? *
    a. No, it does not change with age
    b. Usually increases with age
    c. Usually decreases with age
    d. Don't know

SECTION 5: PRIOR BREAST DENSITY KNOWLEDGE

31. Before we invited you to join the study, have you ever heard of something called breast density?[2 7]
    a. No
    b. Yes

32. Have you ever discussed your own breast density with a health care provider?[2]
    a. No
    b. Yes

    [IF YES]
    What led to your discussion about breast density?[2]
    a. I asked my health care provider about my breast density
    b. My health care provider brought up the topic of density with me
    c. Something else, please specify: [TEXT BOX]

    Do you have dense breasts? [2]
    a. No
    b. Yes
    c. Don't know

    [IF YES] [MULTIPLE RESPONSES ALLOWED]
    Who was it that told you that you have dense breasts?[2]
    a. The health care provider who ordered my mammogram
    b. A radiologist who read my mammogram/my mammogram report
    c. An imaging/x-ray technician (radiographer)
    d. Someone else, please specify: **[TEXT BOX]**

33. Have you heard about breast density from sources other than a health care provider? [2]
    a. No
    b. Yes

    [IF YES] [MULTIPLE RESPONSES ALLOWED]
    From what other sources have you heard about breast density?[2]
    a. Book, magazine, newspaper
    b. Radio or television
    c. Internet
    d. A friend or family member
    e. **[TEXT BOX]**Some other source, please specify:

© 2022 Dolan H et al. *JAMA Network Open.*

SECTION 6: BREAST SCREENING HISTORY

34. Have you ever had a mammogram before? [2]
    a. No
    b. Yes

    [IF YES]

    35. How old were you when you had your first mammogram?[2]
        a. Less than 40 years old
        b. 40-44 years old
        c. 45-49 years old
        d. 50-54 years old
        e. 55-59 years old
        f. 60 years or older
        g. Don't know

    36. How many total mammograms do you think you've had in your lifetime?[2]
        a. 1
        b. 2-4
        c. 5-9
        d. 10 or more

    37. When was your last (most recent) mammogram?[2]
        a. Within the past year
        b. Between one and two years ago
        c. Between two and five years ago
        d. More than five years ago
        e. Don't remember

    38. Where did you have your most recent mammogram?[2]
        a. BreastScreen (publicly funded screening program) centre
        b. Mammography van/unit
        c. Hospital setting (radiology department)
        d. Private screening/medical imaging service
        e. [TEXT BOX]Other, please specify:

    39. Have you ever been called back for additional tests after any mammogram? [2]
        a. No
        b. Yes
        c. I can't remember/don't know

        [IF YES]

        40. How often have you been called back for additional tests? [2]
            a. Only once
            b. Occasionally
            c. Most of the times that I have had a mammogram
            d. Every time that I have had a mammogram

    41. Have you ever had a breast biopsy? [2]
        a. No
        b. Yes
        c. I can't remember/don't know

        [IF YES]

        42. On how many occasions have you had breast biopsies? [2]
            a. 1
            b. 2
            c. 3
            d. 4 or more

43. Are there any questions you have about the information you have read today?
    [Free text]

44. Is there any other information you want to tell us about this survey?
    [Free text]

SECTION 7: DEBRIEF STATEMENT

Title: Australian women's intentions and psychological outcomes related to breast density notification: a randomised experiment

You were a participant in this study which aimed to investigate how women would react to different formats information provision on mammogram results with or without breast density notification.

During the study, you were asked to imagine a scenario in which you receive a letter about your mammogram results after having gone to a routine mammogram screening. You were then asked to complete a series of questionnaires.

You were randomised to receive one of three example letters about mammogram results. These three letters were in different formats.

1. Example standard screening mammogram result only
2. Example standard screening mammogram result AND breast density notification messaging from an existing source
3. Example standard screening mammogram result AND breast density notification messaging that are health literacy sensitive

The purpose of this study was to examine the impact of these different formats of letters on psychological outcomes such as worry and health seeking intentions.

It is important to remember that this study was entirely hypothetical (made up). You did not receive information or advice on your real mammogram results or breast density.

Currently in Australia, BreastScreen services DO NOT measure or report on breast density with the mammogram. This is based on the best available scientific evidence. You can read about BreastScreen Australia's position statement on this matter on the next page.

If you have any further questions regarding the study, feel free to contact the researchers:

Dr Hankiz Dolan, email: hankiz.dolan@sydney.edu.au
Dr Brooke Nickel, email: brooke.nickel@sydney.edu.au

For more information on mammogram, please visit the BreastScreen website in your state:

BreastScreen ACT: https://health.act.gov.au/services-and-programs/women-youth-and-children/womens-health/breast-screening
BreastScreen NSW: http://www.bsnsw.org.au/
BreastScreen Northern Territory: https://nt.gov.au/wellbeing/cancer-services/breastscreennt
BreastScreen Queensland: http://www.breastscreen.qld.gov.au/
BreastScreen South Australia: http://www.breastscreensa.sa.gov.au/
BreastScreen Tasmania:
http://www.dhhs.tas.gov.au/cancerscreening/population_screening_and_cancer_prevention/breast_screening
BreastScreen Victoria: http://www.breastscreen.org.au/

© 2022 Dolan H et al. *JAMA Network Open.*

We thank you for taking time to complete this questionnaire.

SECTION 8: LAY SUMMARY

A summary of the BreastScreen Australian's position statement on breast density

For full position statement, please visit
https://www.health.gov.au/sites/default/files/documents/2020/10/breastscreen-australia-position-statement-on-breast-density-and-screening-breastscreen-australia-2020-position-statement-on-breast-density-and-screening.pdf

Breast density

The breast is made up of glandular tissue, connective tissue and fatty tissue. The glandular tissue and connective tissue are called dense breast tissue. Breast density refers to the relative amounts of dense breast tissues in the breasts.

Dense breast tissue appears white on the mammogram (x-ray) image. Fatty tissue appears black (dark).

Breast cancers appear white on the mammogram image. Dense breast tissue can make it harder to spot the breast cancer on a mammogram.

Having dense breasts can increase your risk of developing breast cancer. However, the increased risk is **smaller** than the risk from other factors, such as family history and genetics.

For example, a woman has a close relative who had breast cancer before having a menopause. Her risk of having breast cancer doubles. A woman carries a BRCA gene mutation. Her risk of having breast cancer is three to six times more than those without the genetic mutation.

It is common for women to have dense breasts. Among women aged 50 or above, around one in three women have dense breasts. Breast density has nothing to do with how breasts look or feel. It is not about the size or firmness of the breasts.

Research on breast density and screening

We know that dense breast tissue makes it harder to spot the cancer on a mammogram. There are many risk factors for developing breast cancer. Having dense breasts is one of them. The other risk factors include being overweight, obesity, drinking alcohol, or whether a woman has had children. Age is the biggest risk factor for breast cancer.

Supplemental testing means after having a mammogram, having additional screening using other methods, such as ultrasound, MRI or 3D mammogram.

To date, there is **NO** scientific evidence to prove that supplemental screening benefits women whose only risk factor is high breast density and who do not have breast symptoms. There is **no evidence** to show that supplemental screening makes these women less likely to die from breast cancer.

If a woman with high breast density has supplemental screening, she might experience some potential harms, including:

- Supplemental screening can be unnecessary and invasive
- Supplemental screening might suggest cancer is present, when in fact there is no cancer in the breast
- Having unnecessary breast biopsies
- Being treated for a condition that will never have caused you symptoms or death
- Having psychological stress due to being diagnosed and treated for cancer
- Spending extra money on additional testing and treatment.
- Extra costs to the health system

Measuring density

There are two ways to measure breast density: by looking at an x-ray image of the breast, or by using a computer software. Both methods have limitations.

Different radiologists might look at the same mammogram and label the breast density differently. The computer software results can also be inconsistent.

There are limitations to the measurement of breast density. A woman might be told she has dense breasts when in fact her breasts should have been categorized as 'not dense'. The woman might become anxious and worried that she might have a missed breast cancer. Or a woman might be told she does not have dense breasts when in fact her breasts should have been categorized as 'dense'. This woman might develop a false sense of security.

There is some scientific evidence that using other tests to screen for breast cancer might pick up cancers that are missed on mammogram. However, the evidence is limited. The benefits of supplemental screening have **not been shown** to outweigh potential risks and harms.

Recommendation

The Standing Committee on Screening recommends that, BreastScreen Australia **SHOULD NOT** routinely record breast density or provide supplemental testing for women with dense breasts. More scientific evidence is needed on how breast density is best assessed and managed.

BreastScreen Australia acknowledges that breast density is an important issue. However, more research is required. BreastScreen Australia supports discussion with women, researchers and service providers. BreastScreen Australia will keep reviewing new evidence.

SECTION 9: FEEDBACK

Thank you for your participation in the survey. Your time and contribution is greatly appreciated.

If you wish to receive feedback about the overall results of the study, please contact the researchers via the contact details outlined in the Participant Information Statement . Alternatively, you can visit the Sydney Health Literacy Lab website https://sydneyhealthliteracylab.org.au/publications/ to check for any relevant publications.

^Validated questions/measures
*****Self-developed questions

© 2022 Dolan H et al. *JAMA Network Open.*

eReferences:

1. Ware Jr JE. SF-36 health survey. 1999
2. Rhodes DJ, Jenkins SM, Hruska CB, et al. Breast Density Awareness, Knowledge, and Attitudes Among US Women: National Survey Results Across 5 Years. *J Am Coll Radiol* 2020;17(3):391-404. doi: 10.1016/j.jacr.2019.11.003 [published Online First: 2019/11/23]
3. Sutton S, Bickler G, Sancho-Aldridge J, et al. Prospective study of predictors of attendance for breast screening in inner London. *Journal of Epidemiology & Community Health* 1994;48(1):65-73.
4. Wellbeing measures in primary health care/the DEPCARE project: report on a WHO meeting, Stockholm, Sweden 12-13 February 1998. Wellbeing measures in primary health care/the DEPCARE project: report on a WHO meeting, Stockholm, Sweden 12-13 February 1998; 1998.
5. Chew LD, Bradley KA, Boyko EJ. Brief questions to identify patients with inadequate health literacy. *health* 2004;11:12.
6. Lipkus IM, Kuchibhatla M, McBride CM, et al. Relationships among Breast Cancer Perceived Absolute Risk, Comparative Risk, and Worries. *Cancer Epidemiology Biomarkers & Prevention* 2000;9(9):973-75.
7. Mahorter SS, Knerr S, Bowles EJA, et al. Prior breast density awareness, knowledge, and communication in a health system-embedded behavioral intervention trial. *Cancer* 2020;126(8):1614-21. doi: 10.1002/cncr.32711 [published Online First: 2020/01/25]

© 2022 Dolan H et al. *JAMA Network Open.*

eTable: Change in Cancer Worry

Cancer Worry	Control (N=480)	WA letter (N=470)	HL letter (N=470)
**Reduce**	155	85	89
**Same**	299	276	272
**Increase**	26	109	109

© 2022 Dolan H et al. *JAMA Network Open.*
