# Supplementary material for: Australian Women’s Intentions and Psychological Outcomes Related to Breast Density Notification and Information: A Randomized Clinical Trial
Source: JAMA Netw Open. 2022 Jun 16;5(6):e2216784. doi: 10.1001/jamanetworkopen.2022.16784 (PMC9204548; doi:10.1001/jamanetworkopen.2022.16784)
Supplement: Supplement 3. — Data Sharing Statement [file jamanetwopen-e2216784-s00.pdf]

Data Sharing Statement

Dolan. Australian Women's Intentions and Psychological Outcomes Related to Breast Density Notification and Information. *JAMA Netw Open*. Published June 16, 2022. doi:10.1001/jamanetworkopen.2022.16784

Data
Data available: No

Additional Information
Explanation for why data not available: In line with the ethics approval, participant data has been made anonymous and aggregated for the purpose of statistical analyses. Findings are reported at the group/condition level rather than the individual participant level.
